# Pharmacokinetics and safety of highly variable nitroglycerin lingual spray (NLS) versus US-licensed nitroglycerin lingual spray reference product (Nitrolingual^®^): a randomized, four-cycle, four-sequence, partially replicated crossover Phase I bioequivalence clinical trial

**DOI:** 10.3389/fphar.2025.1631341

**Published:** 2025-08-28

**Authors:** Yingying Fang, Lu Qi, Yu Wang, Chunyu Han, Qian Zhang, Xiaoyun Liu, Ju Liu, Xiaoqiang Cheng, Yinjuan Li, Yan Li, Mingli Sun, Long Liu, Pu Li, Yingjuan Zhang, Huijuan Liu, Xinghe Wang

**Affiliations:** Department of Phase I Clinical Trial Center, Beijing Shijitan Hospital, Capital Medical University, Beijing, China

**Keywords:** bioequivalence, nitroglycerin lingual spray, pharmacokinetics, safety, early clinical trial

## Abstract

**Purpose:**

In this Phase I study, we aimed to evaluate the pharmacokinetic bioequivalence and the safety of a nitroglycerin lingual spray (NLS) compared with US-approved Nitrolingual in healthy subjects.

**Methods:**

Subjects (N = 56) were randomized 1:1 to receive one 96-mg NLS, US-approved Nitrolingual; safety was assessed for 11 days. Bioequivalence was evaluated using the average bioequivalence method to test whether the 90% confidence intervals (CIs) of the geometric means (NLS vs. US-approved Nitrolingual) for the primary endpoints were within prespecified acceptable ranges (80%–125%).

**Results:**

After the single administration of NLS (test preparation vs. reference preparation) under fasting conditions, the geometric mean ratios (GMRs) of C_max_, AUC_0−t_, and ${\text{AUC}}_{0-\infty }$ were 108.08%, 108.20%, and 110.85%, respectively. For the primary metabolites of NLS (1, 2-GDN), the GMRs of C_max_, AUC_0−t_, ${\text{AUC}}_{0-\infty }$ were 107.68%, 102.28%, and 102.23%, respectively. Similarly, for another metabolite of NLS (1, 3-GDN), the GMRs of C_max_, AUC_0−t_, and ${\text{AUC}}_{0-\infty }$ were 106.56%, 102.05%, and 104.67%, respectively. All 90% CIs for the test/reference AUC ratio and C_max_ ratio were within the acceptable range (80%–125%) for BE, which met the requirements of bioequivalence. No serious adverse events (AEs) occurred, and all AEs were mild and transient.

**Conclusion:**

The bioequivalence of NLS to US-approved Nitrolingual was demonstrated; the safety results of the two study drugs were also similar. These studies provided robust evidence of the pharmacokinetics similarity between NLS and US-approved originator Nitrolingual.

**Clinical Trial Registration:**

http://www.chinadrugtrials.org.cn/index.html, identifier CTR20230675.

## 1 Introduction

Angina pectoris has a high out-of-hospital incidence and high mortality rate. Nitroglycerin plays an important role in prehospital emergency care of patients with angina pectoris; one such example is US-approved Nitrolingual (G. Pohl-Boskamp GmbH & Co. KG), which is authorized for use in the United States and Germany.

Nitroglycerin sublingual spray is of particular interest, which can effectively improve the difficulties in handling, is easy to store and use, and is now a necessary medicine for home use worldwide, especially for the elderly in most European countries. Moreover, the spray formulation, favored as the preferred short-acting agent, could bypass additional disintegration and dissolution steps required for sublingual tablets before absorption (Sanders et al., 1986). A recent study demonstrated that nitrates administered by spray were more efficacious than sublingual nitroglycerin (NTG) tablets in coronary vasodilation for coronary CT angiography (CCTA). A total of 2,024 patients who underwent CCTA at least twice using both spray and tablets were involved in the retrospective study (Kim et al., 2021).

Nitroglycerin lingual spray (NLS) is a US-approved Nitrolingual candidate being developed by SL Pharm. US-approved Nitrolingual belongs to the highly variable drug which demonstrated a high intrasubject variability (≥30%). In a pharmacokinetic (PK) study, when a single 0.8-mg dose of the Nitrolingual Pumpspray was administered to healthy volunteers (n = 24), the mean C_max_ and T_max_ were 1,041 pg/mL and 7.5 min, respectively. Additionally, the mean area under the curve (AUC) in these subjects was 12,769 pg/mL * min (U.S. Food & and Drug Administration, 2018; ECA, 2025). Moreover, the physicochemical characteristics associated with the analysis of NTG, including the rapid metabolism, erratic absorption, relative instability of the analytical samples, variable formulations, and range of data on concentrations following sublingual application, are wide. Thus, it is remarkably difficult to conduct a PK bioequivalence study on NLS. According to the provisions of the Food and Drug Administration (FDA) guidelines, demonstrating PK bioequivalence is key to establishing biosimilarity. A replicate design is recommended to be used, and a reference-scaled BE (RSABE) analytical approach would be applied to specific PK metrics in the BE study (U.S. Food & and Drug Administration, 2021). Thus, we reported the PK data and safety of a single dose of a proposed biosimilar NLS and US-approved Nitrolingual and assessed the bioequivalence of NLS to US-approved Nitrolingual with respect to pharmacokinetics and safety.

## 2 Subjects and methods

### 2.1 Study population

The study was performed at the Beijing Shijitan Hospital-Phase I Clinical Research Center (Beijing, China) from March 2023 to April 2023. This study was registered in the Drug Trial Registration and Information Publication Platform (http://www.chinadrugtrials.org.cn/index.html) as CTR20230675. All subjects were informed regarding the study, and they provided written informed consent prior to their participation. Adult male subjects aged 18–50 years with a body mass index (BMI) between 19.0 kg/m^2^ and 26.0 kg/m^2^ were recruited in the trial. Subjects were required to be in good health without any clinically significant chronic or acute infection. Good health for each subject was determined through a complete medical history review, physical examination, vital sign assessment, 12-lead electrocardiogram (ECG), and clinical laboratory tests, including hematology, electrolytes, liver enzymes, fasting glucose, cholesterol, bilirubin, creatinine, urea, and urine drug testing. All testing was conducted during a 2-week period prior to randomization.

### 2.2 Study design

The trial objective was to establish a single-center, randomized, open, four-cycle, four-sequence, crossover bioequivalence trial and was conducted to assess the pharmacokinetics and safety of NLS and US-approved Nitrolingual following a single sublingual administration in healthy subjects. US-approved Nitrolingual was used in this Phase I clinical study to comply with the US FDA, stating that comparisons must be made to the locally approved reference product.

**FIGURE 1:**
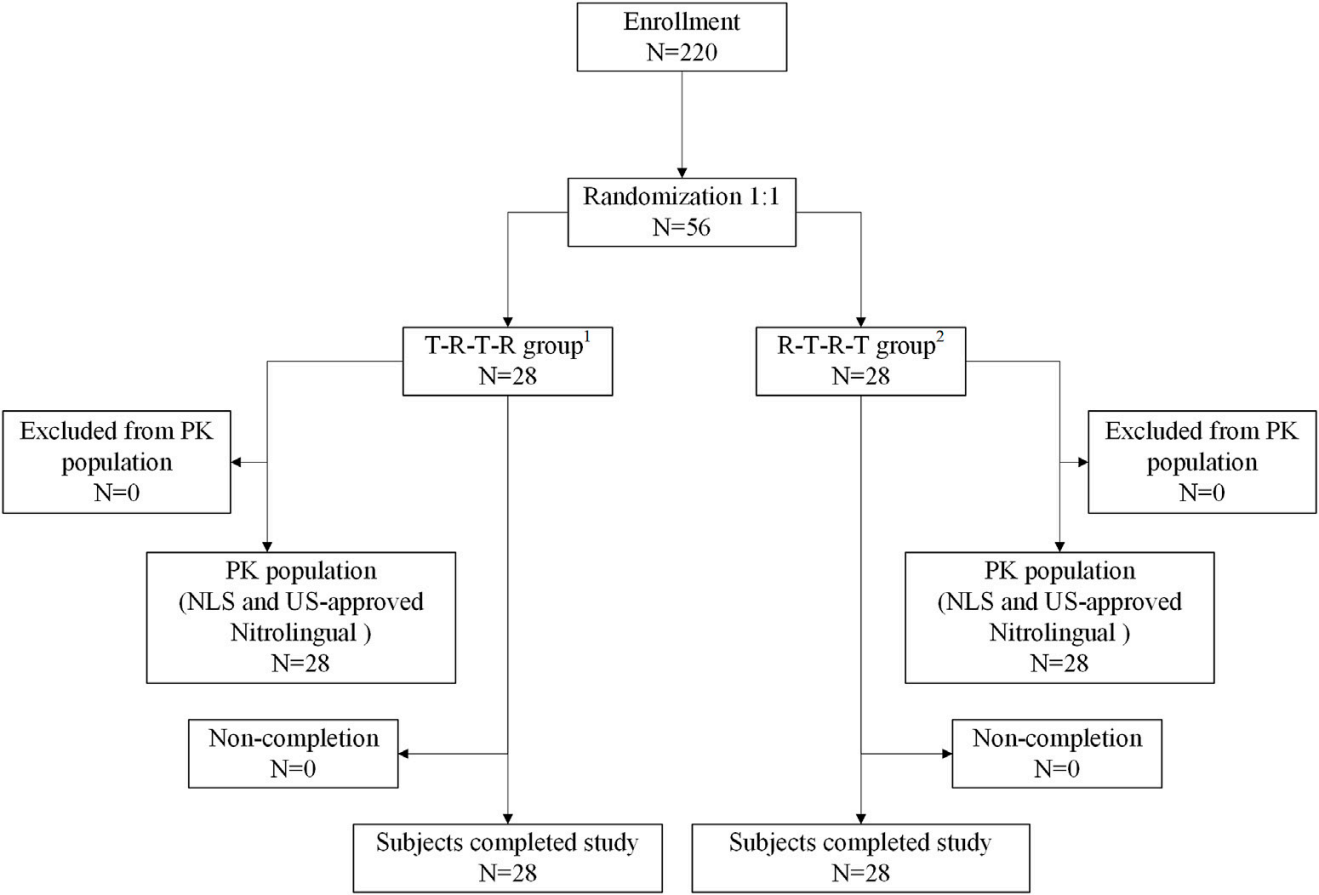
Patient flow. ^1^T-R-T-R group: NLS administration in periods 1 and 3 and US-approved Nitrolingual administration in periods 2 and 4. ^2^R-T-R-T group: US-approved Nitrolingual administration in periods 1 and 3 and NLS administration in periods 2 and 4. Abbreviations: PK, pharmacokinetics; NLS, nitroglycerin lingual spray.

Before the start of the study, the Ethics Committee of Beijing Shijitan Hospital reviewed and approved the study. The study protocol was reviewed and approved by the Institutional Review Board. Each patient was required to read, understand, and sign an informed consent form (ICF) with the latest version before enrolling in the clinical trial. Subjects were enrolled at one site in China, Beijing, stratified by the site and randomized 1:1 to NLS or US-approved Nitrolingual (Figure 1). To follow FDA guidance and demonstrate PK similarity, subjects received one sublingual dose (96mg/0.8 mg) of US-approved Nitrolingual on days 1, 4, 7, and 10 of the study. Single administration of the approved 96-mg dose (96mg/0.8 mg) was deemed to have an acceptable risk/benefit ratio when given to healthy subjects.

### 2.3 Sample size determination

Based on historical and pretest data, an intersubject variability of 20%–50% was assumed for sample size determination. Assuming an intersubject coefficient of variation (CV) of 30%, 45 evaluable subjects per treatment arm were required to provide 80% joint power using PASS V22 (at the α = 0.05 level). To allow for a proportion of 20% non-pharmacokinetics evaluable subjects, 56 subjects were planned to be enrolled. Finally, a total of 56 subjects were randomized in the study.

### 2.4 Study population

Healthy adult male subjects (≥18–≤50 years) who passed a complete medical assessment, had a BMI of ≥19.0–≤26.0 kg/m^2^, and provided informed consent were eligible to enroll in this study.

Key exclusion criteria included previous exposure to a biologic, exposure to restricted drugs within a defined time frame, specific lifestyles (including smoking and alcohol abuse), and an inability/unwillingness to comply with protocol requirements, including extensive blood draws for PK. Subjects were required to be in good health without any clinically significant chronic or acute infection. Good health was determined for each subject through an examination of their medical history, a physical examination, vital sign assignment, urine drug testing, clinical laboratory tests, serology, and electrocardiography. Subjects attended a screening visit and testing less than 14 days before the first dosing of the study drug in period 1.

Upon arrival at the unit in period 1, subjects were randomized 1:1 (using a computer-generated list) to receive a single sublingual administration (0.8 mg) of NLS or US-approved Nitrolingual in each period. There was a wash-out period of at least 2 days between the two study drug periods. In four periods, eligible subjects resided in the clinical unit for at least 24 h prior to dosing.

### 2.5 Drug administration

The subjects were split into three groups of 18–20 subjects each to allow simultaneous dosing and blood sampling. Each subject had a device for drug administration and was trained many times according to the SOP to ensure that the administration was standardized prior to beginning the study. Administered by professionally trained personnel, every action of the administration is given on time according to the prescribed audio. Subjects wore a protective mask before and after dosing to prevent any contamination. They sprayed the dose directly under the tongue and were instructed not to swallow immediately.

### 2.6 Study objectives and endpoints

The primary objective of this study was to evaluate bioequivalence between NTG, 1, 2-glycerol dinitrate (1, 2-GDN), and 1, 3-glycerol dinitrate (1, 3-GDN) of NLS and US-approved Nitrolingual. The secondary objectives were to evaluate the safety and tolerability and other PK parameters of NLS and US-approved Nitrolingual. The primary endpoints were the area under the concentration–time curve (AUC) from time zero to predicted infinity ( ${\text{AUC}}_{0-\infty }$ ), AUC from time zero to the last measurable concentration (AUC_0–t_), and the maximum observed drug concentration in plasma (C_max_). Other PK parameters assessed were terminal elimination half-life of the analysis in plasma (T_1/2_), time from administration to maximum observed concentration of the analysis in plasma (T_max_), and terminal elimination rate constant (λ_z_).

### 2.7 Blood sampling and bioanalytical assays

Pharmacokinetics and safety data were collected from each patient during the study. Initial blood samples were collected at predose and day 1. Subjects subsequently visited the site on 0 min, 1 min, 2 min, 3 min, 4 min, 5 min, 6 min, 7 min, 8 min, 10 min, 12 min, 16 min, 20 min, 30 min, 45 min, 60 min, 90 min, and 120 min after drug administration to provide blood samples for pharmacokinetic testing.

Venous blood was collected in 4-mL heparin sodium anticoagulant blood collection tubes containing 40 μL of 40 mmol/L silver nitrate solution which had been chilled in ice–water mixture for 15 min prior to sample collection to prevent spontaneous denitration of NTG. Immediately after collection, each sample was stored in an ice–water mixture and subsequently centrifuged at 1,700 g for 10 min at 4°C. Plasma was separated from red blood cells and frozen at −80°C until analyzed. Each plasma sample was analyzed in duplicate for the content of NTG, 1, 2-GDN, and 1, 3-GDN. No more than 1 hour elapsed between blood collection and the initial placement of the plasma sample in the refrigerator. In all cases, the analysis was completed within 2 weeks of sample collection.

A validated LC-MS/MS method (Sun et al., 2015; Kim et al., 2018) was used to determine the plasma concentrations of NLS, 1, 2-GDN, and 1, 3-GDN. Liquid chromatography was performed using the LC-40D system (Shimadzu, Japan), whereas the mass spectrometer was the AB SCIEX Triple Quad TM 5500^+^ (Applied Biosystems, United States). The data acquisition software was Analyst 1.7.2 (Applied Biosystems, United States) (Wang et al., 2025). The internal standards included nitroglycerin-D5 (Clearsynth Labs Ltd., lot number: CRC-0557-S-03; chemical purity: 92.20%; isotopic purity: 94.23%), glycerol,1,2-dinitrate-d5 (Quality Control Solutions Ltd., lot number: 26,552; purity: 99.33%), and glycerol,1,3-dinitrate-d5 (Quality Control Solutions Ltd., lot number: 26,553; purity: 98.26%).

NTG, 1, 2-DNG, and 1, 3-DNG were extracted from plasma using liquid–liquid extraction. Quantitative analysis was performed using a triple quadrupole tandem mass spectrometer (Triple Quad TM 5500^+^) operated in a multiple reaction-monitoring (MRM) mode. The plasma concentration range of the standard curve for this analytical method for NTG was 0.05–10.0 ng/mL, and the LLOQ, LQC, MQC, and HQC of the NTG to be measured were 0.05, 0.15, 3.75, and 7.50 ng/mL, respectively. The plasma concentration range of the standard curve for this analytical method for 1,2-GDN was 0.1–20.0 ng/mL, and the LLOQ, LQC, MQC, and HQC of the NTG to be measured were 0.10, 0.30, 7.50, and 15.0 ng/mL, respectively. The plasma concentration range of the standard curve for this analytical method for 1,3-GDN was 0.05–10.0 ng/mL, and the LLOQ, LQC, MQC, and HQC of the NTG to be measured were 0.05, 0.15, 3.75, and 7.50 ng/mL, respectively. Concentrations below this limit were reported as BLQ.

The accuracy deviations of QC samples in NTG ranged from 1.9% to 4.0%, with a precision maximum (%CV) of 7.7%. The accuracy deviations of QC samples in 1,2-GDN ranged from 0.7% to 3.3%, with a precision maximum (%CV) of 7.9%. The accuracy deviations of QC samples in 1,3-GDN ranged from 1.9% to 4.0%, with a precision maximum (%CV) of 5.8%.

Chromatographic separation of the samples was performed on Thermo Scientific, Aquasil C18 (5 μm, 2.1 × 100 mm), with a mobile phase containing 0.025 mmol/L ammonium chloride aqueous solution and 0.025 mmol/L ammonium chloride acetonitrile solution at a flow rate of 0.6000 mL/min.

All samples were tested using the same technique, to ensure consistency in terms of methods used. WinNonlin8.3 software (Certara^®^, Princeton, NJ, United States) was used to perform the pharmacokinetic parameter calculations.

### 2.8 Safety evaluations

Adverse events (AEs) were recorded at each visit during the study. Laboratory tests, vital signs, an electrocardiogram, and clinical examination were also performed during the study. AEs were classified by the System Organ Class and Preferred Term using the Medical Dictionary for Regulatory Activities (MedDRA) version 17.0. AEs were assessed for severity and related to the study drug. All incidences of AEs during the study were collected, documented, and reported according to the protocol instructions. Any AEs occurring within 11 days of study drug administration were considered to be on-treatment; AEs after that period were not recorded. A total of 64 patients experienced mild AEs, whereas only four patients experienced moderate AEs. A total of 98 events were judged to be related to the drug treatment: 46 with NLS and 52 with the originator. No relevant differences in the severity, type, or pattern of AEs were observed between the two treatments. None of the reported AEs was considered severe, significant, or serious. Overall, the most commonly observed AEs occurred with similar frequencies with both treatments; most of these AEs were expected with the use of NLS or US-approved Nitrolingual.

### 2.9 Statistical analysis

The analysis strategy used in this study was in line with that recommended by the FDA, EMA, and the ICH. The sample size was calculated so that the power to conclude two-way PK bioequivalence (with an acceptable range of 80%–125%, one-sided significance level of 5%, and taking into account the two comparisons) was approximately 90%, assuming no treatment differences, and approximately 80% for a 5% treatment difference (ratio scale). The assumed variability was based on pretest and literature data on the approved versions of Nitrolingual.

This study was powered at 90% for a sample size of 56 subjects for both pharmacokinetic objectives. The safety population was defined as the group of subjects who received at least one dose of the study medication. The per-protocol (PP) analysis population included all subjects who received the study drug, provided evaluable pharmacokinetic profiles, and completed the study without a protocol violation. The primary pharmacokinetic analyses were based on this PP population.

The ratios of the geometric means (NLS vs. US-approved Nitrolingual) and their two-sided 90% confidence intervals (CIs) were provided for all primary and secondary PK endpoints; the analysis was conducted using an analysis of covariance (ANOVA) model on the similarity of ${\text{AUC}}_{0-\infty }$ compared to NLS and US-approved Nitrolingual. The analysis of the Pratt–Wilcoxon test for the two groups was used for statistical comparisons. Equivalence of NLS and US-approved Nitrolingual was to be concluded if the 90% CIs for the ratios of the geometric means of the primary pharmacokinetic parameter met the prespecified acceptance criteria (80%–125%). All PK parameters were analyzed descriptively, with geometric coefficients of variation given to measure dispersion. The analysis was performed using SAS^®^ version 9.2 (SAS Institute, Inc., Cary, North Carolina).

## 3 Results

### 3.1 Subject demographics and baseline characteristics

Overall, 220 subjects were screened for this study. Of the 220 subjects, 56 (41 male and 15 female subjects) were randomized, received four doses of study medication, and were included in the safety analysis. Subject demographics and baseline characteristics were comparable between the two groups (Figure 1). The 56 patients included in the safety analysis had a mean (±standard deviation) age of 33.91 ± 6.90 years [median 33.50 (range 21.00–47.00)], height of 167.15 ± 7.97 cm [median 168.65 (range 153.70–181.40)], weight of 64.96 ± 7.33 kg [median 65.05 (range 51.40–81.90)], and BMI of 23.22 ± 1.72 kg/m^2^ [median 23.60 (range 19.30–26.00)].

### 3.2 Subject disposition

There were no subjects excluded from the pharmacokinetic analysis and safety analysis. Therefore, a total of 56 subjects were included in the pharmacokinetic analysis and the safety analysis (Figure 1).

### 3.3 Pharmacokinetics for NTG

**FIGURE 2:**
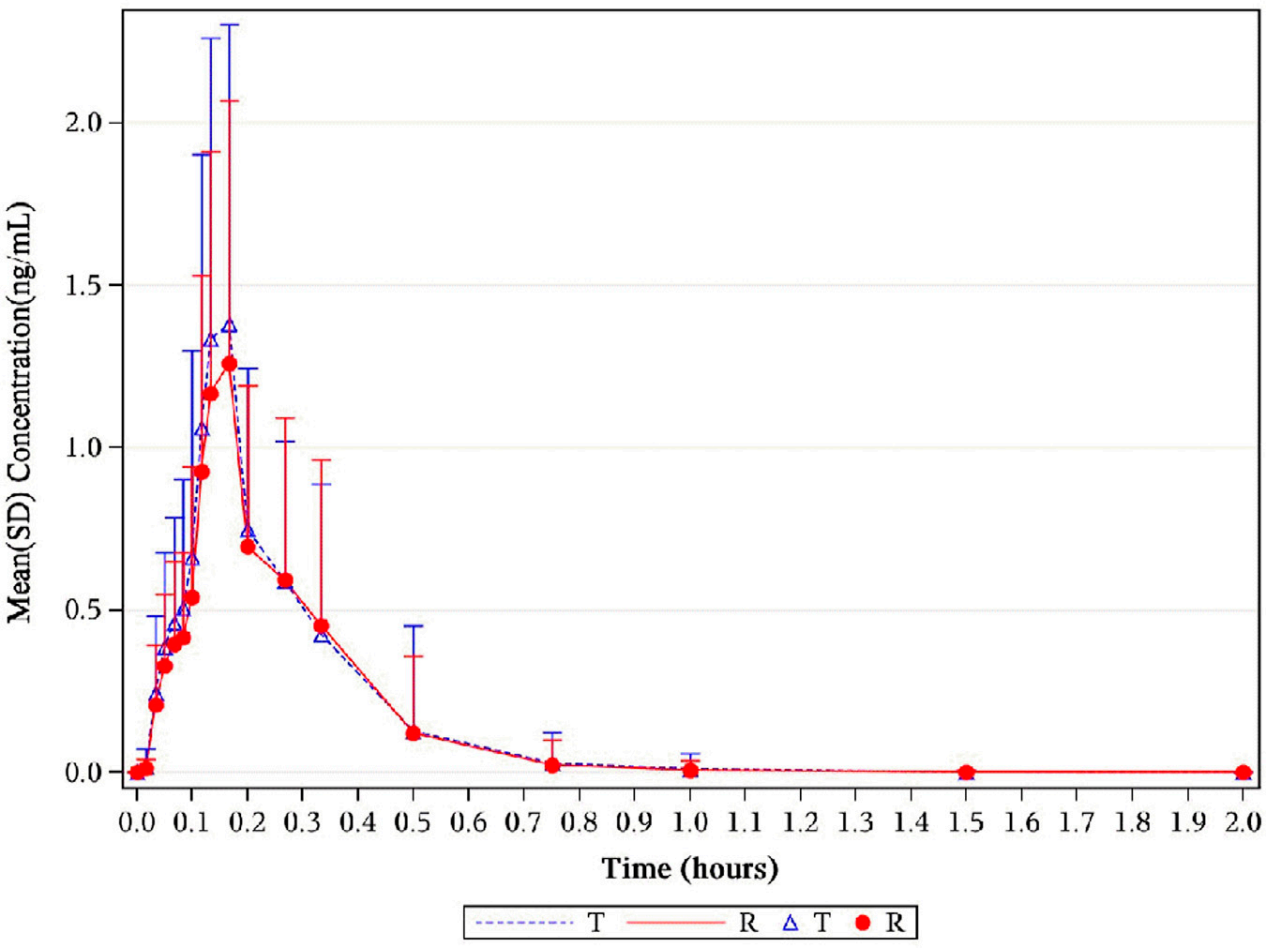
Mean NTG plasma concentration–time profiles in healthy subjects following a single 0.8-mg sublingual for NLS and US-approved Nitrolingual. Results of the statistical comparison of primary PK endpoints corroborated the similarity over the entire profiling period. Abbreviations: NTG, nitroglycerin; NLS, nitroglycerin lingual spray; PK, pharmacokinetics.

**FIGURE 3:**
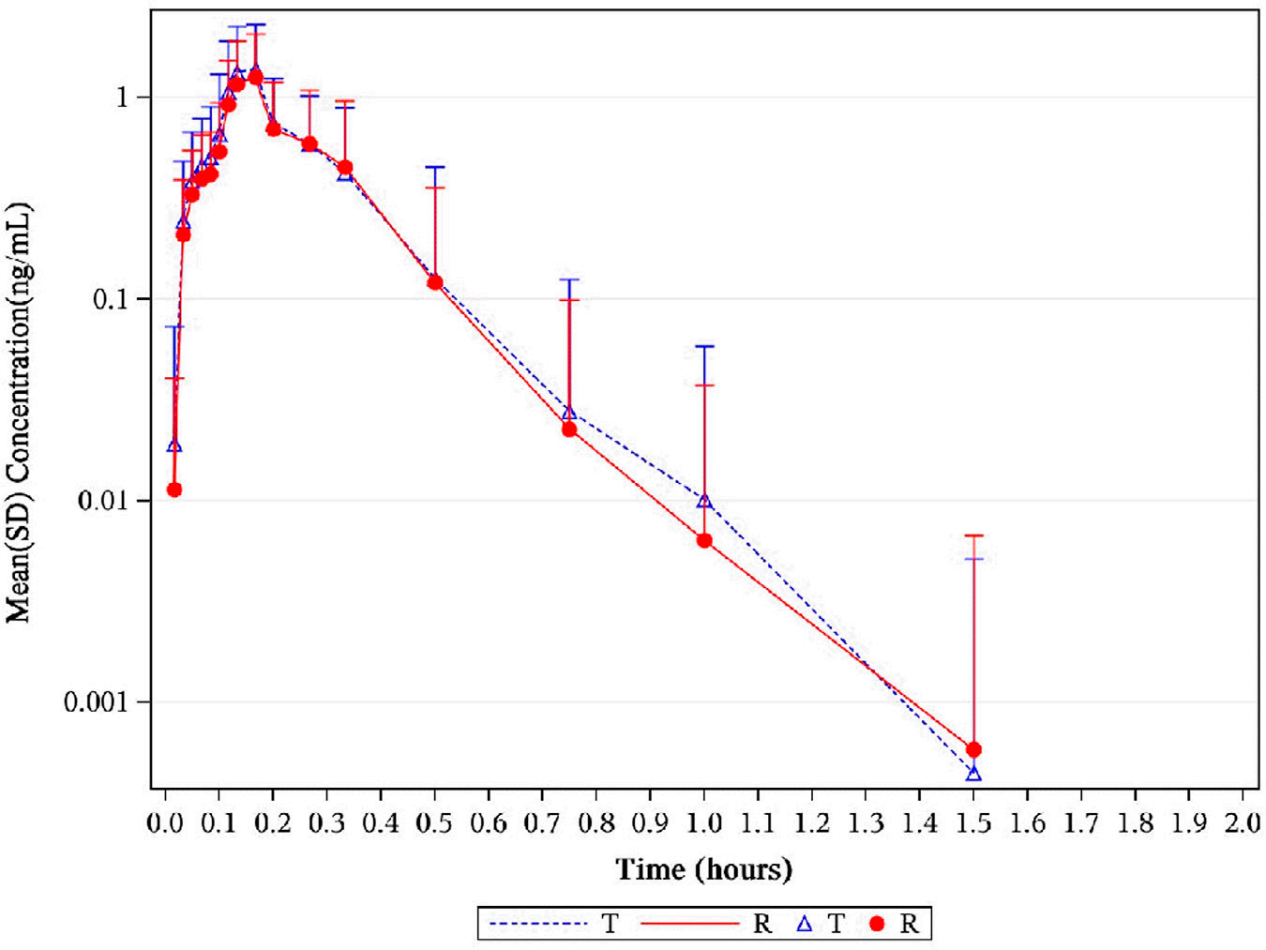
Mean serum concentration curves of NTG for each group on a semi-logarithmic scale. The mean serum concentration–time profiles of NTG between NLS and US-approved Nitrolingual were highly similar. Abbreviations: NTG, nitroglycerin; NLS, nitroglycerin lingual spray.

The median T_max_ was 0.17 h for NLS and US-approved Nitrolingual. Unadjusted geometric mean C_max_ levels were 1.56 ng/mL and 1.44 ng/mL for NLS and US-approved Nitrolingual, respectively. Unadjusted geometric mean ${\text{AUC}}_{0-\infty }$ levels were 0.31 ng*h/mL and 0.29 ng*h/mL for NLS and US-approved Nitrolingual, respectively. An overview of the geometric mean PK parameters is provided in Table 1. Mean plasma concentration–time profiles of NTG for all two treatments were comparable over the entire profiling period (Figures 2, 3).

**TABLE 1:** An overview of the geometric mean PK parameters of NTG, 1,2-GDN, and 1,3-GDN in healthy subjects after dosing.

PK parameter	Geometric mean ± SD					
	NTG of NLS (N = 56)	NTG of US-approved Nitrolingual (N = 56)	1,2-GDN of NLS (N = 56)	1,2-GDN of US-approved Nitrolingual (N = 56)	1,3-GDN of NLS (N = 56)	1,3-GDN of US-approved Nitrolingual (N = 56)
T_max_ (h)	0.17 (0.07,0.5)	0.17 (0.08,0.5)	0.33 (0.13,1.5)	0.33 (0.17,1.03)	0.50 (0.2,1.5)	0.50 (0.17,1.03)
C_max_ (ng/mL)	1.56 ± 0.95	1.44 ± 0.84	4.22 ± 1.38	3.96 ± 1.44	1.50 ± 0.55	1.41 ± 0.55
AUC_0-t_ (h*ng/mL)	0.29 ± 0.22	0.26 ± 0.20	3.97 ± 0.98	3.89 ± 0.98	1.45 ± 0.46	1.42 ± 0.44
${\text{AUC}}_{0-\infty }$ (h*ng/mL)	0.31 ± 0.22	0.29 ± 0.24	5.14 ± 3.56	4.78 ± 1.56	2.42 ± 6.50	1.70 ± 0.59
λ_z_ (1/h)	8.95 ± 4.08	9.27 ± 4.11	1.24 ± 0.39	1.15 ± 0.44	1.22 ± 0.37	1.13 ± 0.40
T_1/2_(h)	0.11 ± 0.11	0.13 ± 0.33	0.81 ± 1.20	0.79 ± 0.65	1.12 ± 4.00	0.78 ± 0.70

Abbreviations: PK, pharmacokinetics; SD, standard deviation; NTG, nitroglycerin; 1,2-GDN, 1,2-glycerol dinitrate; 1,3-GDN, 1,3-glycerol dinitrate; NLS, nitroglycerin lingual spray; T_max_, time from administration to maximum observed concentration; C_max_, maximum observed drug concentration; AUC_0–t_, area under the concentration–time curve from time zero to the last measurable concentration; ${\text{AUC}}_{0-\infty }$ , area under the concentration–time curve from time zero to predicted infinity; λ_z_, terminal elimination rate constant; T_1/2_, terminal elimination half-life.

Point estimates of the adjusted GMR and the 90% CIs for the ratios of the primary PK endpoints for the comparison of NLS and US-approved Nitrolingual were 110.85% (102.04%–120.42%) for ${\text{AUC}}_{0-\infty }$ , 108.20% (100.53%–116.46%) for AUC_0–t_, and 108.08% (99.50%–117.39%) for C_max_ (Table 2). Bioequivalence of NTG of NLS and US-approved Nitrolingual was declared for the comparisons. The statistical comparison of the PK parameters showed that the 90% CIs of the adjusted GMRs were all within 80%–125% limits and as such supported similarity between NLS and US-approved Nitrolingual (Figure 8).

**TABLE 2:** Summary of PK parameters of NTG, 1,2-GDN, and 1,3-GDN in healthy subjects after a single dose of NLS and US-approved Nitrolingual.

PK parameter	N_T_	NLS GLSM	N_R_	US-approved Nitrolingual GLSM	GMR %	90% CI (%)	CV (%)
NTG							
C_max_ (ng/mL)	112	1.34	112	1.24	108.08	99.50–117.39	29.50
AUC_0-t_ (h*ng/mL)	112	0.23	112	0.21	108.20	100.53–116.46	25.33
${\text{AUC}}_{0-\infty }$ (h*ng/mL)	103	0.26	94	0.23	110.85	102.04–120.42	32.88
1,2-GDN							
C_max_ (ng/mL)	112	4.00	112	3.71	107.68	102.36–113.27	23.41
AUC_0-t_ (h*ng/mL)	112	3.84	112	3.76	102.28	99.28–105.36	15.76
${\text{AUC}}_{0-\infty }$ (h*ng/mL)	102	4.63	94	4.53	102.23	96.71–108.06	17.80
1,3-GDN							
C_max_ (ng/mL)	112	1.41	112	1.32	106.56	100.39–113.12	27.41
AUC_0-t_ (h*ng/mL)	112	1.38	112	1.35	102.05	98.56–105.66	17.65
${\text{AUC}}_{0-\infty }$ (h*ng/mL)	100	1.70	94	1.63	104.67	97.22–112.70	17.35

Abbreviations: PK, pharmacokinetics; NTG, nitroglycerin; 1,2-GDN, 1,2-glycerol dinitrate; 1,3-GDN, 1,3-glycerol dinitrate; NLS, nitroglycerin lingual spray; N_T_, number of subjects in the NLS group; N_R_, number of subjects in the US-approved Nitrolingual group; GLSM, generalized least squares method; GMR, geometric mean ratio; CI, confidence interval; CV, coefficient of variation; C_max_, maximum observed drug concentration; AUC_0–t_, area under the concentration–time curve from time zero to the last measurable concentration; ${\text{AUC}}_{0-\infty }$ , area under the concentration–time curve from time zero to predicted infinity.

### 3.4 Pharmacokinetics for 1, 2-GDN

The distribution of the time of maximum plasma concentration (T_max_) of each group is similar among the groups. The serum concentration reached a maximum between 0.13 h and 1.5 h after dose administration in all cases, with a median time to C_max_ (T_max_) of 4.22 ng/mL (0.33 h) and 3.96 ng/mL (0.33 h) for 1, 2-GDN of NLS and US-approved Nitrolingual, respectively. The other mean values of pharmacokinetic parameters including ${\text{AUC}}_{0-\infty }$ , AUC_0–t_, T_½_, and elimination rate constant (λz) were similar among the two treatment groups (Table 1).

**FIGURE 4:**
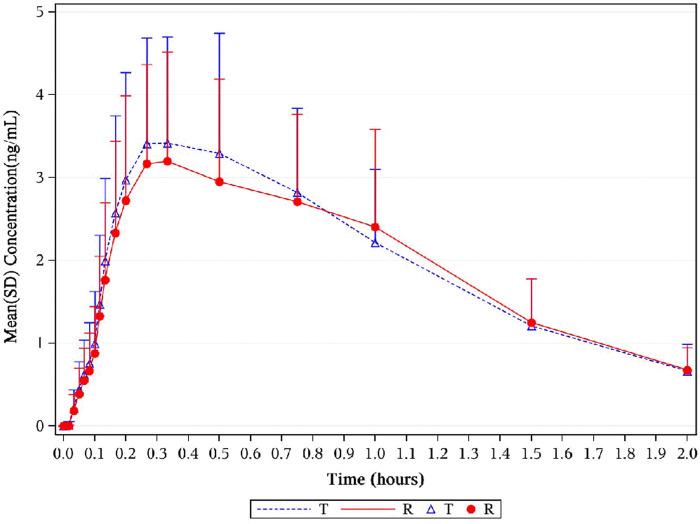
Mean plasma concentration–time profiles of 1,2-GDN in healthy subjects. Results of the statistical comparison of primary PK endpoints corroborated the similarity over the entire profiling period. Abbreviations: 1,2-GDN, 1,2-glycerol dinitrate; PK, pharmacokinetics.

**FIGURE 5:**
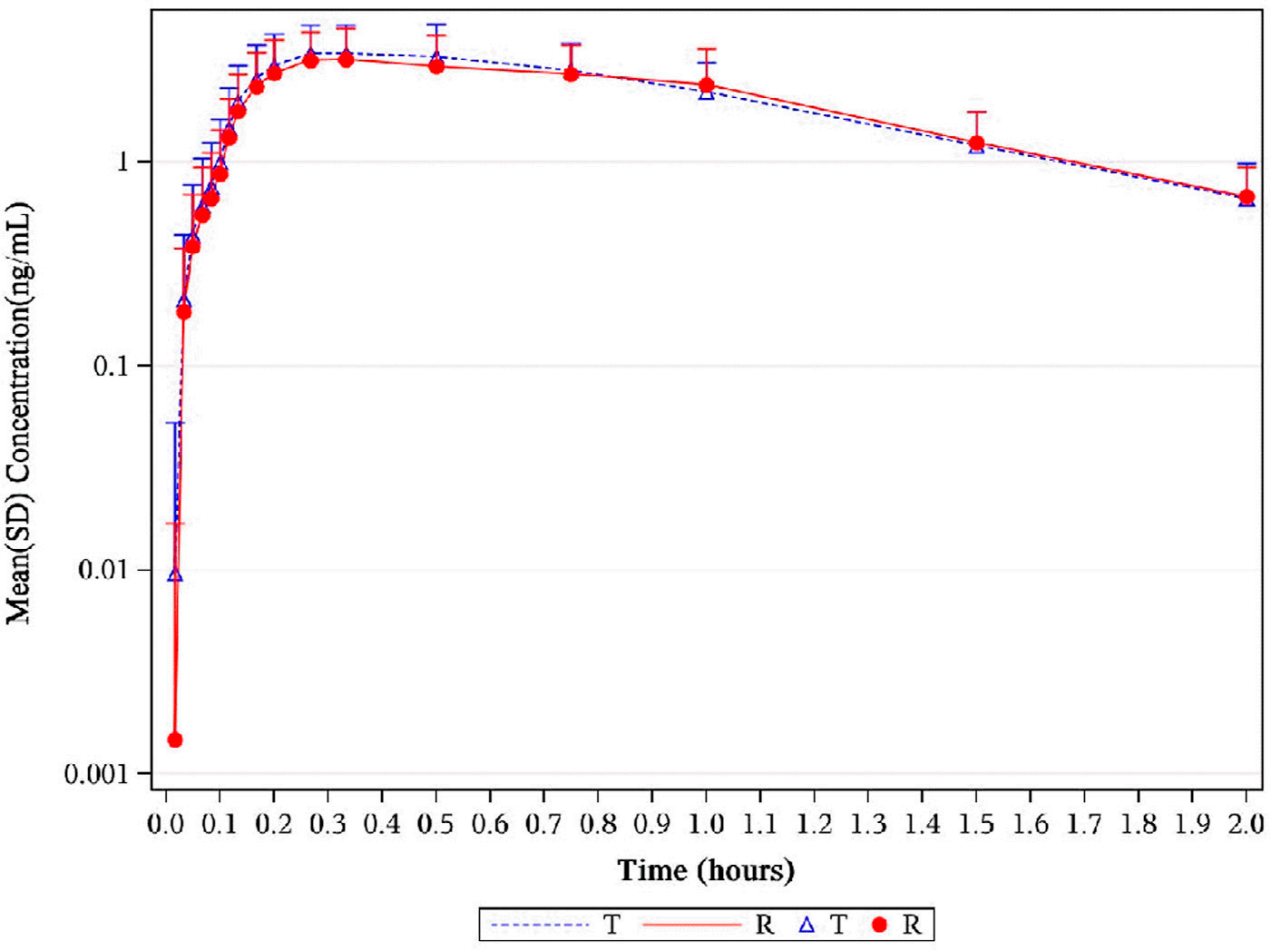
Mean serum concentration curves of 1,2-GDN for each group on a semi-logarithmic scale. The mean serum concentration–time profiles of 1,2-GDN between NLS and US-approved Nitrolingual were highly similar. Abbreviations: 1,2-GDN, 1,2-glycerol dinitrate; NLS, nitroglycerin lingual spray.

The point estimates of the adjusted GMRs and the 90% CIs for the ratios of the primary PK endpoints for the comparison of active metabolites (1, 2-GDN) of Nitrolingual and US-approved Nitrolingual were 102.23% (96.71%–108.06%) for ${\text{AUC}}_{0-\infty }$ , 102.28% (99.28%–105.36%) for AUC_0–t_, and 107.68% (102.36%–113.27%) for C_max_ (Table 2). Corresponding CIs for the GMRs of the relative endpoints (90% CI) were all within the prespecified acceptance range of 80%–125% (Figures 4, 5, 8).

### 3.5 Pharmacokinetics for 1, 3-GDN

**FIGURE 6:**
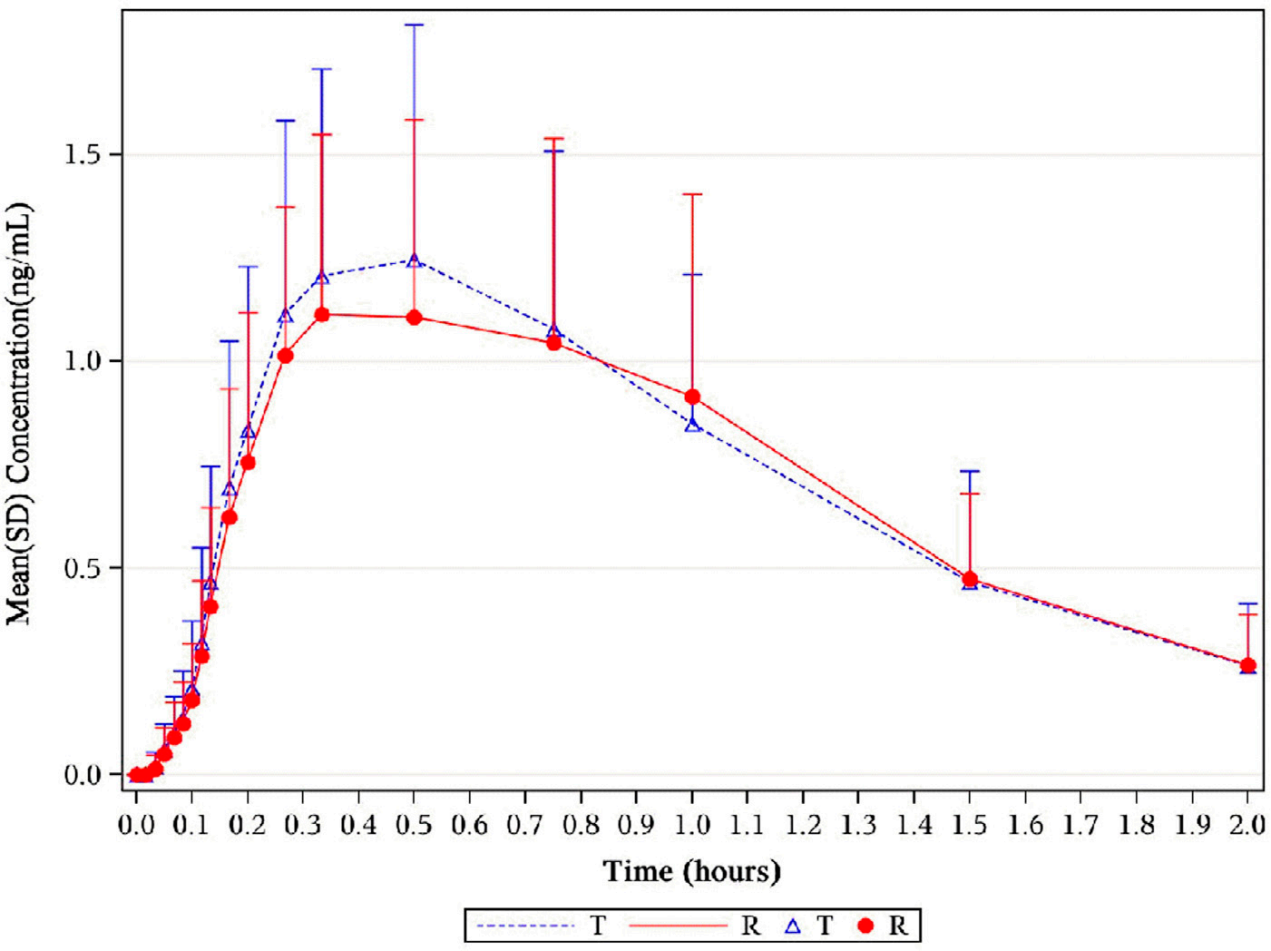
Mean plasma concentration–time profiles of 1,3-GDN in healthy subjects. Results of the statistical comparison of primary PK endpoints corroborated the similarity over the entire profiling period. Abbreviations: 1,3-GDN, 1,3-glycerol dinitrate; PK, pharmacokinetics.

**FIGURE 7:**
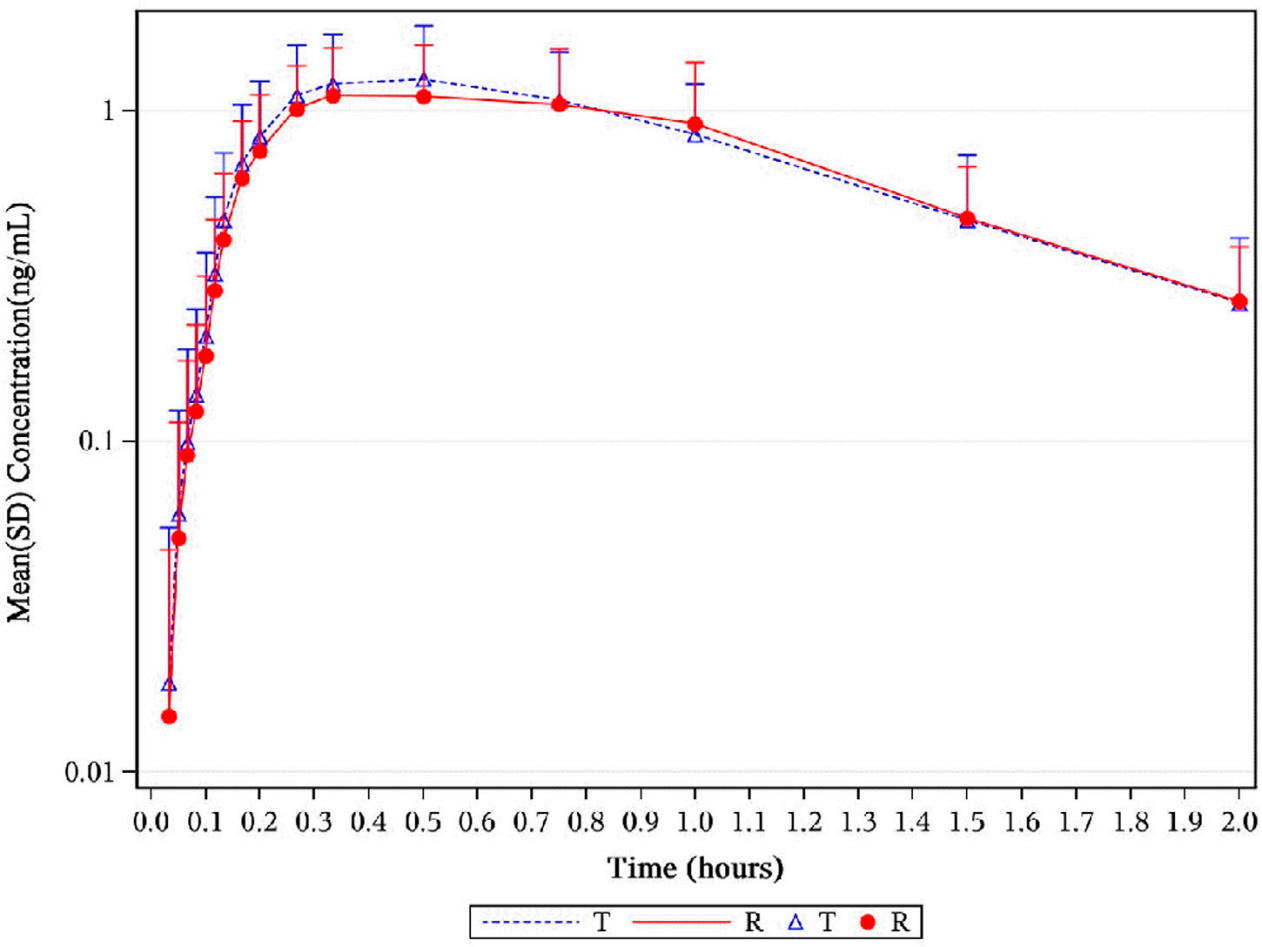
Mean serum concentration curves of 1,3-GDN for each group on a semi-logarithmic scale. Mean serum concentration–time profiles of 1,3-GDN between NLS and US-approved Nitrolingual were highly similar. Abbreviations: 1,3-GDN, 1,3-glycerol dinitrate; NLS, nitroglycerin lingual spray.

**FIGURE 8:**
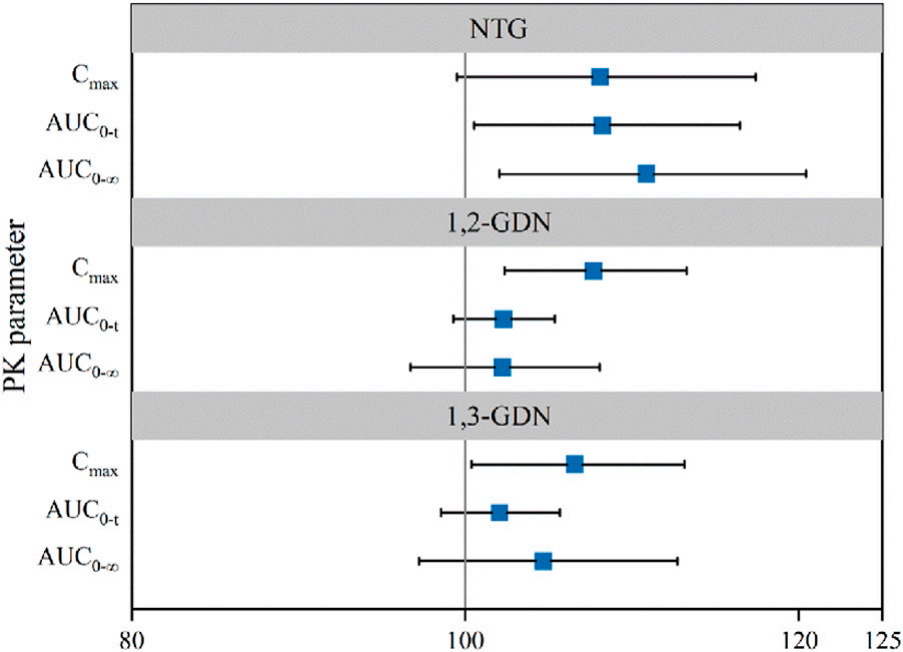
Forest plot presenting point estimate and 90% confidence intervals for primary PK parameters for NLS and US-approved Nitrolingual (bioequivalence was declared if the 90% confidence intervals were within prespecified acceptance ranges of 80%–125%). Abbreviations: PK, pharmacokinetics; NTG, nitroglycerin; 1,2-GDN, 1,2-glycerol dinitrate; 1,3-GDN, 1,3-glycerol dinitrate; NLS, nitroglycerin lingual spray; C_max_, maximum observed drug concentration; AUC_0–t_, area under the concentration–time curve from time zero to the last measurable concentration; ${\text{AUC}}_{0-\infty }$ , area under the concentration–time curve from time zero to predicted infinity.

The median time at which T_max_ was detected was 0.50 h (median values) and 0.50 h (median values) for 1, 3-GDN of NLS and US-approved Nitrolingual, respectively. Unadjusted geometric mean C_max_ levels were 1.50 ng/mL and 1.41 ng/mL for 1, 3-GDN of NLS and US-approved Nitrolingual, respectively. Unadjusted geometric mean ${\text{AUC}}_{0-\infty }$ levels were 2.42 and 1.70 ng*h/mL for 1,3-GDN of NLS and US-approved Nitrolingual, respectively (Table 1). The point estimates of the adjusted GMR and the 90% CIs for the ratios of the primary PK endpoints for the comparison of active metabolites (1,3-GDN) of Nitrolingual and US-approved Nitrolingual were 104.67% (97.22%–112.70%) for ${\text{AUC}}_{0-\infty }$ , 102.05% (98.56%–105.66%) for AUC_0–t_, and 106.56% (100.39%–113.12%) for C_max_ (Table 2). Mean plasma concentration–time profiles of 1, 3-GDN for all two treatments were comparable over the entire profiling period (Figures 6, 7). The 90% CIs for the adjusted GMRs of NTG to reference product analyses for the primary PK endpoints were all within the prespecified acceptance range of 80%–125% (Figure 8).

### 3.6 Safety

The incidence of subjects experiencing at least one AE was similar across the two treatment groups; however, it was slightly lower in the NLS group [25 (44.6%)] than that in the US-approved groups [31 (55.4%)]. There was no serious adverse event (SAE) during the study (Table 3).

**TABLE 3:** Overview of adverse events.

Characteristic	Statistic	NLS (N = 56)	US-approved Nitrolingual (N = 56)	Total (N = 56)
Number of subjects with at least one				
AE	n (%)	25 (44.6)	31 (55.4)	35 (62.5)
SAE	n (%)	0 (0)	0 (0)	0 (0)
AE severity				
I	n (%)	21 (37.5)	20 (35.7)	35 (62.5)
II	n (%)	0 (0)	1 (1.8)	1 (1.8)
AE with relationship with study drug				
Related	n (%)	25 (44.6)	30 (53.6)	35 (62.5)
Unrelated	n (%)	2 (3.6)	3 (5.4)	5 (8.9)

Abbreviations: NLS, nitroglycerin lingual spray; AE, adverse event; SAE, severity adverse event.

The most common adverse drug reaction (ADR) with subject incidence rates ≥2% by treatment are presented in Table 4. Overall, the commonly reported ADRs (reported in ≥10% of subjects) were decreased diastolic blood pressure [11 (19.6%) vs. 10 (17.9%)], elevated heart rate [6 (10.7%) vs. 6 (10.7%)], headache [5 (8.9%) vs. 10 (17.9%)], and dizzy [6 (10.7%) vs. 5 (8.9%)].

**TABLE 4:** Most common adverse drug reactions reported by ≥ 2% of subjects in any treatment group, after a single dose of NLS and US-approved Nitrolingual.

	NLS (N = 56)		US-approved Nitrolingual (N = 56)		Total (N = 56)	
SOC/PT	Frequency	N (%)	Frequency	N (%)	Frequency	N (%)
Total	44	25 (44.6)	49	30 (53.6)	93	35 (62.5)
Investigations	27	21 (37.5)	28	19 (33.9)	55	28 (50.0)
Decreased diastolic blood pressure	13	11 (19.6)	15	10 (17.9)	28	15 (26.8)
Elevated heart rate	7	6 (10.7)	8	6 (10.7)	15	7 (12.5)
Decreased blood pressure	3	2 (3.6)	1	1 (1.8)	4	3 (5.4)
Nervous system disorders	11	10 (17.9)	19	13 (23.2)	30	19 (33.9)
Headache	5	5 (8.9)	14	10 (17.9)	19	13 (23.2)
Dizzy	6	6 (10.7)	5	5 (8.9)	11	10 (17.9)
Gastrointestinal disorders	4	3 (5.4)	0	0 (0)	4	3 (5.4)
Nause	3	3 (5.4)	0	0 (0)	3	3 (5.4)
Cardiac disorders	2	2 (3.6)	2	2 (3.6)	4	4 (7.1)
Tachycardia	2	2 (3.6)	2	2 (3.6)	4	4 (7.1)

Abbreviations: NLS, nitroglycerin lingual spray; SOC, System Organ Class; PT, preferred term.

## 4 Discussion

The PK and safety bioequivalence between NLS and US-approved Nitrolingual was evaluated in a randomized, open, four-cycle, four-sequence, crossover bioequivalence trial which was conducted in 220 healthy participants recruited from young adults. A total of 56 subjects were randomized and completed the study.

PK bioequivalence of NLS is difficult to accomplish because both all-in-one drug and equipment, and the physicochemical characteristics associated with the analysis of NTG, including the rapid metabolism, erratic absorption, relative instability of the analytical samples and variable formulations, and range of data on concentrations following sublingual application, are wide (Hashimoto and Kobayashi, 2003). Moreover, relationships among concentrations of nitroglycerin, its metabolites, and resultant hemodynamic effects are complex. No single explanation for these findings is completely satisfactory (Curry et al., 1993). It is clear from a recent study that not all sprays result in equivalent bioavailability. Thus, the design aspects of the standard operating procedures (SOPs) of both administration and sample processing should be strictly limited.

It is strikingly interesting whether NLS and US-approved Nitrolingual are highly variable drugs (HVDs). It is well known that the disposition of NTG is extremely variable. This is indicated by the CV reported from earlier studies on the disposition of the drug that NTG belongs to HVD. Variation in C_max_ has ranged from 16% to 87%, whereas Noonan and Benet reported variabilities of 43% and 74% for T_max_ and AUC, respectively (Noonan and Benet, 1985). S. W. Sanders et al. observed an approximately 50% CV of NTG in the parameters measured in the study (Sanders et al., 1986). As we all know, HVDs have a risk of bioequivalence and are difficult to study, even when the reference formulation itself is used for comparison. Interestingly, in the study, the PK metrics of NTG C_max_ (29.50%), AUC_0−t_ (25.33%), and ${\text{AUC}}_{0-\infty }$ (32.88%) in US-approved Nitrolingual were less than the results in the previous studies. The CV% of the PK metrics of 1, 2-GDN and 1, 3-GDN were less than 30%.

Reasons for different CV% of NTG may be attributed to factors affecting variation. To begin with, 70% of the variation comes from drug disposition factors. Drug disposition factors include absorption, drug metabolism, and excretion. A fully replicated design in the trial may be to minimize drug disposition factors. In addition, 30% of the variation from the drug formulation, study execution anomalies, and subject factors were strictly performed in the trial.

During the trial, it was extremely important to ensure the uniformity and standardization of the four dosing procedures for the same subject and sample processing, which might affect intra-individual variability to some extent. On the one hand, the key points to make sure the same doses (0.8 mg NLS) are the following: pre-spray before administration, uniformity of body position before administration, dosing steps of administration in seconds, and avoiding swallowing within 5 min after drug administration. On the other hand, the time window of blood collection before 10 min was approximately 30 s. Both blood collection tubes and cryopreservative tubes were precooled, and a stabilizer was added in advance.

The key PK parameter values of nitroglycerin were concordant with prior studies of nitroglycerin in the same formulation, site of administration (sublingual administration), and dosage (0.8 mg). Specifically, relative studies reported C_max_ values of 1.04 ng/mL (T) and 1.66 ng/mL (R), whereas this study observed 1.24 ng/mL (T) and 1.34 ng/mL (R). Similarly, AUC_0−tlast_ values were 0.21 h*ng/mL (T) and 0.25 h*ng/mL (R) in the literature versus 0.23 h*ng/mL (T) and 0.21 h*ng/mL (R) herein. In addition, recent reports documented T_max_ of 0.125 h (7.5 min); our results yielded 0.17 h (almost 10 min) (de Mey et al., 1995; U.S. Food & and Drug Administration, 2018). The concentrations of 1,2-GDN and 1,3-GDN align with existing data from comparable nitroglycerin studies (de Mey et al., 1995). Consequently, these data collectively validate the reliability of the study conclusions.

Safety and tolerability results were comparable between NLS and US-approved Nitrolingual. Safety results were similar between the study drugs, with no clinically meaningful differences observed across the two treatment groups. The AEs, which were reported most frequently (probably, possibly, or unlikely related to treatment), were a decrease in blood pressure after sublingual administration. Only minor side effects were recorded during the study.

Overall, pharmacokinetic equivalence was demonstrated for the comparisons (NLS versus US-approved Nitrolingual). The 90% CIs for the GMRs of all primary PK parameters (for comparisons of NLS with US-approved Nitrolingual) were within the prespecified acceptance ranges (80%–125%), demonstrating PK bioequivalence. Additionally, the statistical comparison of secondary and other PK parameters supported the similarity between NLS and US-approved Nitrolingual. Sublingual administration of 0.8 mg of NLS and US-approved Nitrolingual was found to be safe and well tolerated in this study. There were no remarkable differences between the treatment groups in safety and tolerability parameters.

This study has several limitations that should be acknowledged. First, the sample size was relatively small. This would limit the ability to fully characterize inter-individual variability in PK parameters and safety profiles. Second, the exclusion of special populations (e.g., adolescents, children, and elderly individuals) precludes extrapolation of the current findings to these groups. Future studies are warranted to assess the safety and efficacy of NLS in populations with distinct metabolic or physiological conditions.

## 5 Conclusion

NLS was shown to be highly similar to US-approved Nitrolingual in terms of pharmacokinetic equivalence and safety in healthy subjects.

## Data Availability

The original contributions presented in the study are included in the article/supplementary material; further inquiries can be directed to the corresponding author.
